# Galangin-Loaded Gold Nanoparticles: Molecular Mechanisms of Antiangiogenesis Properties in Breast Cancer

**DOI:** 10.1155/2023/3251211

**Published:** 2023-02-16

**Authors:** Malik H. Qaddoori, Hanady S. Al-Shmgani

**Affiliations:** Department of Biology, College of Education for Pure Sciences (Ibn Al-Haitham), University of Baghdad, Baghdad, Iraq

## Abstract

Angiogenesis is important for tissue during normal physiological processes as well as in a number of diseases, including cancer. Drug resistance is one of the largest difficulties to antiangiogenesis therapy. Due to their lower cytotoxicity and stronger pharmacological advantage, phytochemical anticancer medications have a number of advantages over chemical chemotherapeutic drugs. In the current study, the effectiveness of AuNPs, AuNPs-GAL, and free galangin as an antiangiogenesis agent was evaluated. Different physicochemical and molecular approaches have been used including the characterization, cytotoxicity, scratch wound healing assay, and gene expression of *VEGF* and *ERKI* in MCF-7 and MDA-MB-231 human breast cancer cell line. Results obtained from MTT assay show cell growth reduction in a time- and dose-dependent aspect; also, in comparison to individual treatment, a synergistic impact was indicated. CAM assay results demonstrated galangin-gold nanoparticle capacity to suppress angiogenesis in chick embryo. Additionally, altering *VEGF* and *ERKI* gene expression was recorded. Taken together, all the results can conclude that galangin-conjugated gold nanoparticles can be a promising antiangiogenesis supplemental drug in breast cancer treatment.

## 1. Introduction

As it is well-known nowadays, breast cancer is one of the major causes of death in the world. It accounts for more than 10% of all new instances of cancer in women [1]. Several ways have been developed to deliver medications selectively to cancer lesions. The majority of them depend on certain biological features of the tumor microenvironment, including angiogenesis, an acidic pH, and an overexpression of cell membrane antigens [2], which are taken advantage of in order to acquire access to cancer cells [3]. Chemotherapy, radiation, antihormonal therapy, and antibody therapy all have common side effects that can have a significant impact on daily routines and well-being [4]. Alternative natural biological cancer treatment can mitigate these effects and bring them down to an adaptable level.

Flavonoids are produced in plants as secondary metabolite compounds. Due to their strong antioxidant properties, flavonoids have drawn researchers' interest and have been employed in several investigations to determine whether they might have a positive impact on a variety of diseases [5, 6]. Many studies have revealed that flavonoids can have potent anti-inflammatory and anticancer effects. By focusing on many cancer pathways, including cell metabolism, migration, adhesion, and apoptosis, flavonoids have been shown to reduce carcinogenesis in different cancer cell types [7, 8]. Galangin (3,5,7-trihydroxyflavone, a polyphenol flavonoid is predominantly obtained from several medicinal plants, has been shown to have good pharmacological effects, such as anti-inflammatory, different organ body protection, antidiabetic, and anticancer effects. Also, galangin has been demonstrated to promote autophagy and initiate programmed cell death in cancer cells [9–11]. It has been reported that galangin at high concentrations (500, 1000, and 2000 mg/kg) has no impact on male and female rats either on body weight or mortality nor does it cause inflammation [12].

Nanomedicine has developed into a cutting-edge approach to cancer treatment and a captivating diagnostic tool. It is possible, to recent advancements in the usage of nanoparticles (NPs), to create a safe and very effective method of delivering anticancer drugs to the target tissues [13, 14]. It has been established that the physicochemical characteristics of AuNPs, including the charge, size, and surface chemistry, affect their toxicity [15, 16]. Under both cases of chronic and nonchronic exposure circumstances, Falagan-Lotsch et al., [17] assessed the enduring impacts of AuNPs with various forms and surface coatings.

Angiogenesis can be simply defined as the formation of new blood vessels, it is involved in important normal physiological processes. Antiangiogenic chemicals, on the other hand, are used to produce hypoxia in cancer therapy. Through the development of collateral blood arteries, many growth factors (such as VEGF-A or bFGF) improve blood flow in ischemic tissues [18]. Studies have proven that AuNPs have antiangiogenic properties, which are accomplished by blocking the endothelial cell migration, proliferation, and tube formation [19]. Flavonoid nanoparticles have shown promise in the near future as a cancer treatment, according to both in vitro and in vivo investigations. Flavonoid nanoparticles' anticancer efficacy is linked to apoptosis and antiproliferation, blocking the cancer cells' cell cycle, controlling the host's immune system, or having anti-inflammatory effects. The antitumor effect of medications used in cancer therapy can also be supported by flavonoid nanoparticles, either by improving the antitumor effect or by lowering toxicity of pharmaceuticals [20].

Chorioallantoic membrane assay (CAM) is frequently used in research to assess the tissue's angiogenic potential and response to various biomarkers [21]. Zwadlo-Klarwasser et al. [22] hypothesize that the different materials' diverse chemical compositions may account for how differently they can influence the CAM's angiogenic response.

The identification of new effective and safe chemical as molecular leads for innovative cancer medications is becoming more and more important due to the rising incidence of cancer worldwide. Thus, the current study investigated how the flavonoid galangin loaded on AuNPs could inhibit angiogenesis both *in vitro* in cancer breast cell lines and *in ovo* by controlling *VEGF* and *ERKI* gene expression.

## 2. Materials and Methods

### 2.1. Reagents

Roswell Park Memorial Institute-1640 medium (RPMI-1640), fetal bovine serum, penicillin, and streptomycin trypsin-EDTA (Capricorn Scientific, USA) were used. GoTaq® 1-Step RT-qPCR System, MgCL_2_, Nuclease-Free Water, and Quantifluor RNA System were from Promega (USA), while crystal violate, ethidium bromide, and dimethyl sulfoxide (DMSO) were obtained from Sigma (St. Louis. USA) and TRIzol Reagent from Thermo Scientific (USA). Primers were from Macrogen (Korea), Chloroform LiChrosolv (Germany), and RNA Extraction Kit from Promega (USA). MCF-7 and MDA-MD-231 breast cancer cell lines were obtained from Biotechnology Research Center, University of Al-Nahrain, Baghdad, Iraq. 3-day old chicken embryo eggs were purchased from a local poultry field, Diala, Iraq. This protocol was approved by the committee of ethics in the University of Baghdad 6S/267 in 26/2/2021.

### 2.2. Preparation of Gold Nanoparticles

Preparation of Au nanoparticles was carried out following the standard citrate-reduction Turkevich method [23]. Briefly, 20 ml of 1% trisodium citrate dihydrate (Na_3_C_6_H_5_O_7_∙2H_2_O) was added to 200 ml of 1 mM HAuCl_4_∙3H_2_O, while the mixture was boiling for 10 min and allowed to develop win red color. Then, the mixture was left at room temperature to cool down before being centrifuged at 13,000 rpm for 15 min to remove extra citrate; the pellet was resuspended in distal water and wrapped with aluminum foil and kept at 4°C.

### 2.3. Conjugation of Galangin on Gold Nanoparticle

Galangin was conjugated on AuNPs following Al-Dulimi et al.'s [24] method with some modifications. Briefly, galangin (C_15_H_10_O_5_) was dissolved in dimethyl sulphoxide (0.05% DMSO) and added with heating to AuNPs at a final concentration (25 *μ*l/ml). After cooling to room temperature, the conjugated galangin-gold nanoparticles (AuNPs-GAL) were centrifuged at 13000 rpm for 15 min to remove unconjugated galangin.

### 2.4. Characterization of Prepared Nanoparticles

A UV-vis spectrophotometer (Shimadzu-1650, Japan) was used to carry out scanning spectrum analysis. A Shimadzu (Japan-made) spectrometer with an attenuated total spectral range of 4000-400 cm^−1^ and a resolution of 4 cm^−1^ was used to perform the FTIR analysis. X-ray diffraction was used to measure the prepared nanoparticle crystalline state (XRD-6000, Shimadzu, Japan). To assess the size and shape of synthesized NPs, a field emission scanning electron microscopy (FESEM) study in conjunction with an EDAX team and transmission electron microscopy (TEM, Tecnai G2 20s-TWIN, China) was conducted. To assess the stability of nanoparticles, Brookhaven Zeta PALS equipment (Milton Keynes, UK) evaluated zeta potentials and particle size.

### 2.5. *In Vitro* Anticancer Studies

#### 2.5.1. Cytotoxicity Assay

MTT test was used to determine the cytotoxicity of AuNPs-GAL on the two interesting breast cancer cells [25]. Briefly, 10^4^ cells/well were seeded for 24 h to achieve a confluent monolayer. Various concentrations (10, 25, 50, 75, and 100 *μ*M) of AuNPs, AuNPs-GAL, and galangin were added followed by adding 10 *μ*l of MTT dye then incubated at 37°C for 4 h. The stain was then removed, and 100 *μ*l of DMSO in each well was added and incubated for another 5 min. Absorbance was read at 575 nm, and the following calculation was used to calculate the percentage of cell growth inhibition:

(1) $$\begin{array}{c} \text{Cell growth inhibition}\left(\%\right)=\frac{\text{OD}\left(\text{control}\right)-\text{OD}\left(\text{samples}\right)}{\left(\text{control}\right)\text{OD}}\times 100\%, \end{array}$$

where OD is the optical density.

#### 2.5.2. Quantification of *VEGF* and *ERKI* Gene by RT-PCR

Total RNA samples were extracted from MCF-7 and MDA-MB-231 breast cancer cell line using TRIzol Reagent Kit (Sigma-Aldrich, UK). All work steps were done in accordance with the manufacturer's instructions. Trypsinized cultured cells were collected as a pellets and lysed by adding 250 *μ*l of lysing solution with 2-mercaptoethanol (1%). Then, they were centrifugated at 13000 rpm for 5 min to remove the debris. Ethanol (70%) was added to samples at 1 : 1 ratio before putting the samples to the RNeasy column. Samples were digested at room temperature by buffer/DNase I at a ratio of 70 : 10 for 15 min, followed by a washing step and centrifugation. RNA concentration was determined using an ND-1000 NanoDrop spectrometer (Thermo Scientific, UK). cDNA was produced using a kit of high-capacity cDNA reverse transcription (Promega, USA) according to the manufacturer's procedure. Primers were designed using BLAST-NCBI software (Table 1), and for normalization, GAPDH was reserved as a housekeeping gene. The threshold cycles (Ct) were measured and calculated using the Livak-ΔΔCt method using the StepOnePlus v2.0 software, and the final value was produced using 2-ΔΔCt.

#### 2.5.3. Migration Assay by Wound Healing Procedure

In vitro scratch experiment was used to observe the impact of AuNP, AuNPs-GAL, and galangin (50, 100 *μ*M) on cell-cell communication and migration at 0, 24, and 48 h, following Okamoto et al.'s 2014 protocol with some modifications. Using a sterile 200 *μ*l pipette tip, the monolayer cells were scratched. Cells migrated into the wound surface were photographs at 4x and 10x magnification, and ImageJ software was used for image analysis where the gap width (*μ*m) of the open wound area for each image was calculated [26].

#### 2.5.4. Chorioallantoic Membrane (CAM) Chick Assay

Using the CAM assay, the test compound as antiangiogenesis was investigated according to Balakrishnan et al. [27]. Groups of ten fertilized chicken eggs were incubated at a temperature of 37°C and a relative humidity of 60-80%. Each shell's square window opened after 86 h of incubation. The regions between preexisting vessels were covered with filter paper discs that had been saturated with AuNPs, AuNP-GAL, and galangin at various concentrations 0, 50, and 100 *μ*M and PBS as control group, and the embryos were then incubated for a further 24 h. Photographed CAMs in each test group were analyzed using ImageJ software to assess the percentage of vascular density and vascular length density. Three runs of the assay were performed.

#### 2.5.5. Statistical Analysis

All data were analyzed using SPSS programme (version 23.0). Analysis of variance (ANOVA) followed by LSD test was used to assess significant difference and accepted at *P* ≤ 0.05. Values were demonstrated as mean ± SD.

## 3. Results and Discussion

### 3.1. Preparation of AuNPs and AuNPs-GAL

**Figure 1:**
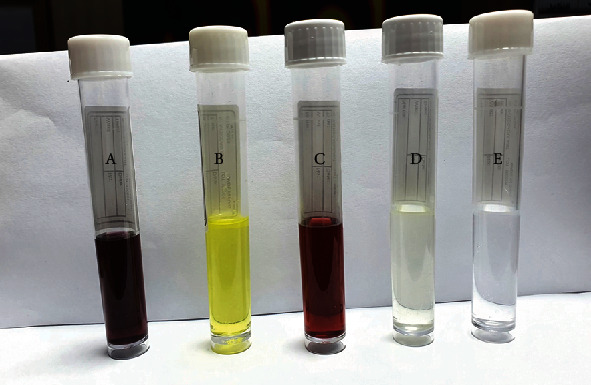
Color changes during the production of gold nanoparticles (GNPs) and galangin- (GAL-) loaded process. (a) AuNPs-GAL, (b) GAL, (c) AuNPs, (d) tetrachloroauric acid trihydrate solution (HAuCl_4_·3H_2_O), and (e) trisodium citrate dihydrate (Na_3_Ct∙2H_2_O).

As shown in Figure 1, there has been no reduction of the tetrachloroauric acid (HAuCl_4_∙3H_2_O); after being combined with the trisodium citrate (Na_3_C_6_H5O_7_∙2H_2_O) as a reducing and capping mediator for the NPs, color changes from yellowish to red indicating AuNP formation. Galangin was then loaded to AuNP solution gradually with heating causing the change of red color to dark wine red.

Trisodium citrate is used in the process to quickly convert any Au^3+^ ions in the solution to Au^+^ ions, which can subsequently be disproportionate to produce metallic gold atoms [28]. The subsequent reduction of Au^+^ ions by these metallic gold atoms in the atom's electron double layer subsequently uses these atoms as nucleation sites, causing the particles to form from the metallic gold nucleus. Trisodium citrate acts to promote the deposition of gold onto the already created nucleation points rather than the formation of new nucleation point [29]. As a result of the electrostatic interaction between the cationic amine group of galangin and the anionic carboxyl of AuNPs, galangin was successfully loaded onto the NPs.

### 3.2. Characterization of AuNPs and AuNPs-GAL

**Figure 2:**
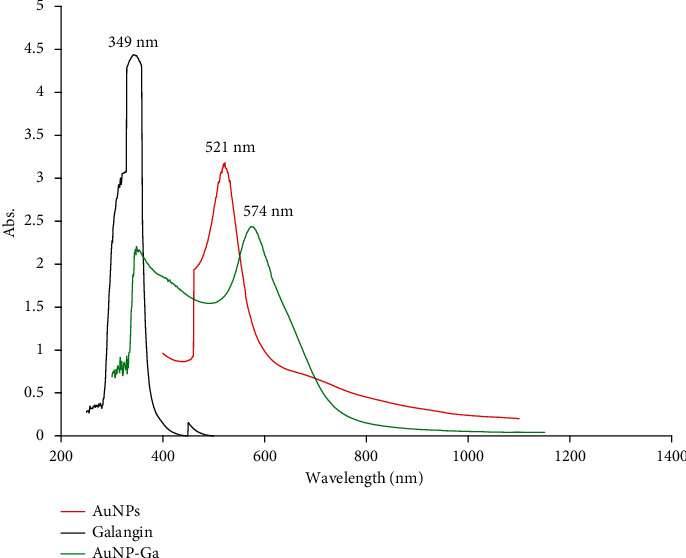
UV-vis spectrum analysis: (1) galangin (black line), (2) AuNPs (red line), and (3) AuNPs-GAL (green line).

Figure 2 shows the absorption spectra of AuNPs at *ʎ*max 521 nm, which may be caused by the small, spherical NPs being excited by the surface plasmon. This study's UV-vis spectroscopy results were used to validate that the AuNPs were synthesized and conjugated to GAL. The absorbance of AuNPs-GAL was demonstrated at *λ*max ~574 nm and this proves that the drug molecules might perhaps adhere to the AuNP surfaces and change their photophysical characteristics. The slight red shift from 522 to 574 nm was attributed to the layer that changed the refractive index around the AuNPs, showing that the AuNP surface modification was successful in achieving the aim [30]. While GAL absorbance was seen at 349 nm, in general, in the UV-visible range, flavonoids show two main absorption bands. The A ring portion (benzyl system, band 2) is represented by the absorptions in the 240-280 nm range, whereas the B ring portion is represented by those in the 320-385 nm range (cinnamoyl system, band 1) [31]. Moreover, it has been suggested that the vibrations in the tune associated with light wave of both electrons in metal NPs and the plasmon resonance (SPR) are responsible for this phenomenon [28].

### 3.3. Fourier Transform Infrared Spectroscopy Analysis (FTIR)

**Figure 3:**
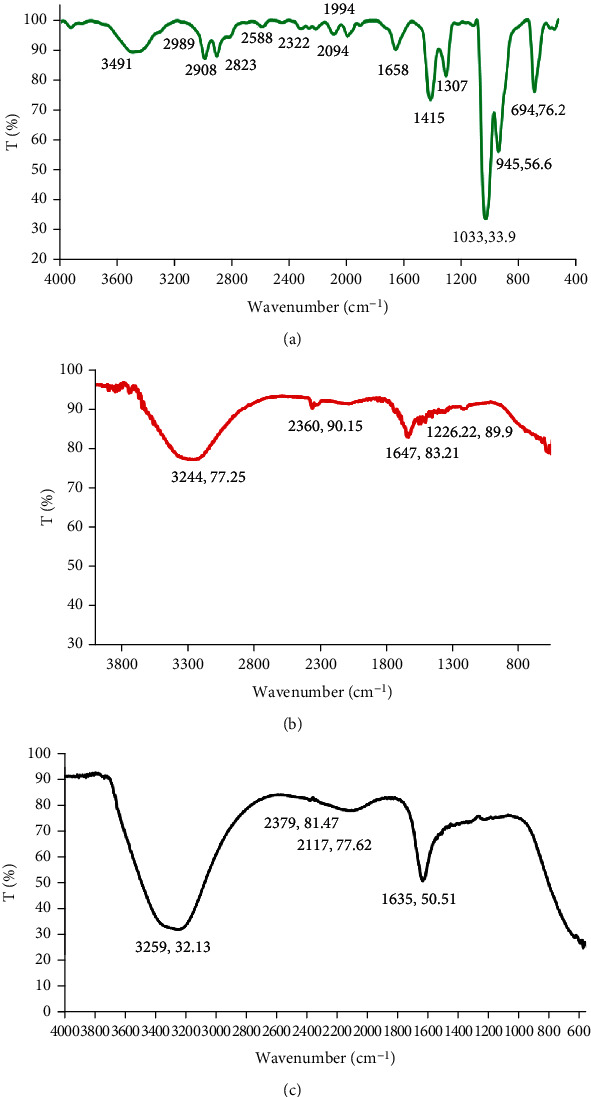
FT-IR spectra of (a) galangin (green line), (b) AuNPs (red line), and (c) AuNPs-GAL (black line).

The functional groups present in the GAL, AuNPs, and AuNPs-GAL were characterized by FTIR. As shown in Figure 3, the FTIR spectrum of synthesized AuNPs presents a broader peak observed at 3244.27 cm^−1^ assigned as O-H stretching of alcohol band accompanied with a medium-intensity H-bounded vibration, two more peaks were found, three-bond C-C stretching vibration of alkyne was at the peak at 2360.87 cm^−1^, and the band located at 1647.21 cm^−1^ allocated two C-C stretching of alkene. The peak at 516.92 cm^−1^ was C-Br stretching strong vibration of alkyl halide. The peak at 486.06 and 462.92 and 432.05 cm^−1^ corresponds to C-I. C-C was a strong vibration located at the fingerprint region that interprets existing single bonds such as C-X. The bands at 1226.92 cm^−1^ are for the C-O carboxylic group. FTIR spectra for AuNPs-GAL showed a peak at 3259 cm^−1^ for the alcohol-OH group which indicates the possible dative bonding between Au and –OH.

New peaks were generated and appeared in FT-IR spectra of AuNPs-GAL, which were not AuNP spectra; the NH group at 3259.84 cm^−1^ indicated galangin presence on the AuNPs. These results are consistent with other studies [32] who showed that the protonated amine group might interact electrostatically with the anionic of AuNPs, potentially forming hydrogen bonds. Additionally, the chemical bonds and functional groups were assessed using FTIR analysis, and any chemical interactions that developed in the polymer as a result of the drug accompaniments during nanoparticle manufacturing were characterized.

### 3.4. X-Ray Diffraction Technique (XRD)

**Figure 4:**
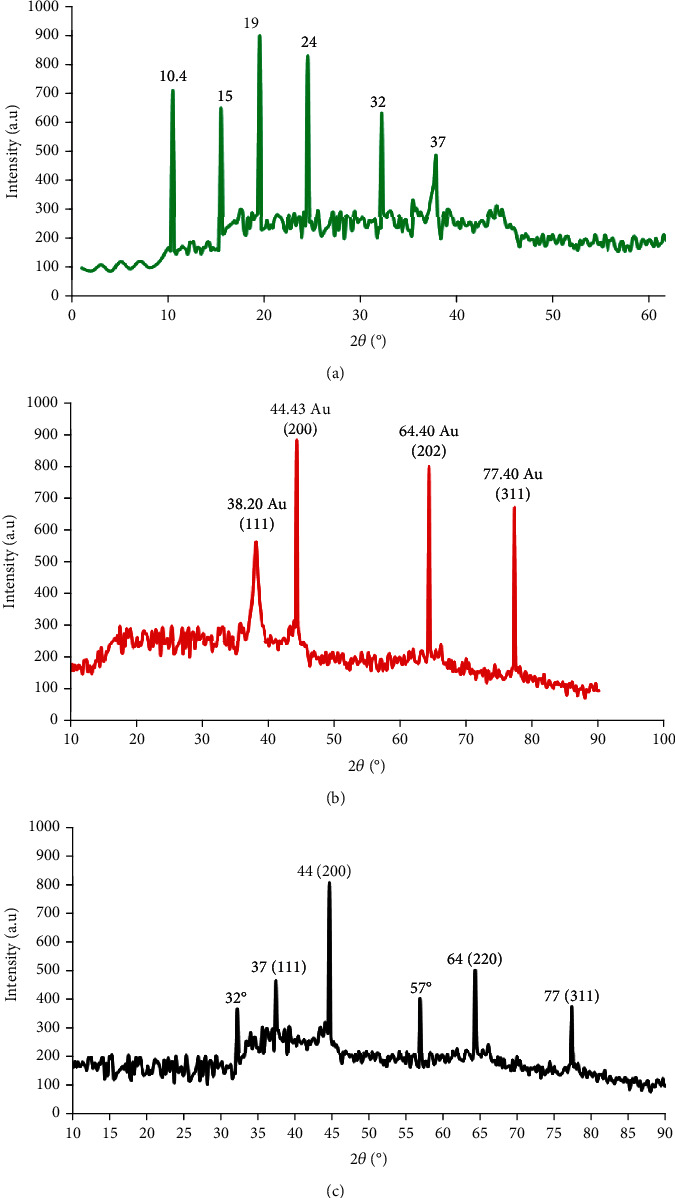
X-ray diffraction: (a) galangin (green line), (b) AuNPs (red line), and (c) AuNPs-GAL (black line).

The crystal structures were demonstrated by XRD. Peaks were founds at 2*θ* values of 38°, 44°, 64°, and 77° corresponding to the FCC gold reflections of 111, 200, 220, and 311 in the XRD spectra of AuNPs as shown in Figure 4. The samples have the very intense peak 200 of FCC AuNPs. Peak intensity showed a strong crystal structure in AuNPs. The modest size of the crystal and confirmation of the polycrystalline face-centered cubic structure are responsible for the broad diffraction peaks [33]. Furthermore, the AuNPs showed all of the conjugate diffraction peaks, demonstrating the structure stability of the AuNPs in the presence of GAL. This data can explained by the expansion of the organic coating as the GAL conjugation process had no impact on the metallic core size.

### 3.5. SEM and TEM Electron Microscopy

**Figure 5:**
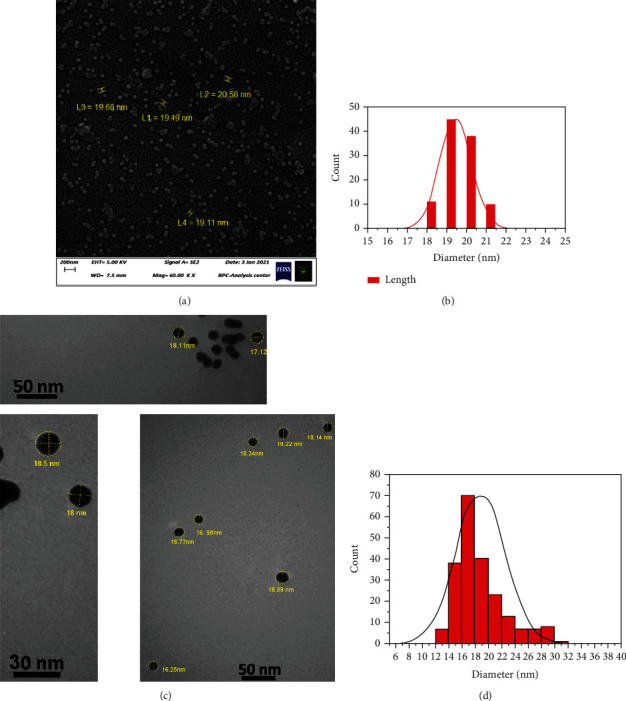
(a, b) Show scanning electron microscopic images for AuNPs-GAL and particle size distribution histogram. (c, d) Show transmission electron microscopic images for AuNPs-GAL and particle size distribution histogram.

The FESEM images displayed a spherical, smooth, and almost homogenous structure of the AuNPs-GAL that were formed with an average size of 19.17 ± 1.61 nm as shown in Figures 5(a) and 5(b). Results of transmission electron microscopy images showed AuNP-GAL average size at 19.45 ± 0.5 nm (Figures 5(c) and 5(d)).

### 3.6. MTT Cytotoxicity Test

**Figure 6:**
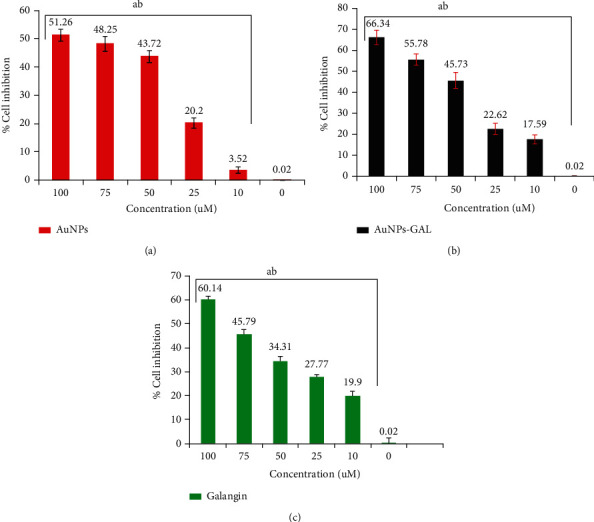
Cytotoxicity and growth cell inhibition determined by MTT assay on MCF-7 human breast cancer cell growth exposed to (a) AuNPs, (b) AuNPs-GAL, and (c) GAL at different concentrations (10, 25, 50, 75, and 100 *μ*M). Statistical analysis indicates (a) significant difference compared to the control group and (b) significant difference between groups. Data are mean ± SD from three independent experiments.

Results from the MTT assay give a precise explanation about some underlying mechanisms cells take in response to toxicity. As compared to GAL or AuNPs in the current study, the synthesized AuNPs-GAL showed a higher level of anticancer activity against MCF-7 and MDA-231 cells, with dose-dependent cellular damage. The recorded cell inhibition for MCF-7 cells at the highest concentration 100 *μ*M was 66.34% for AuNPs-GAL, 60.14% for GAL, and 51.26% for AuNPs. At 10 *μ*M, the growth inhibitions for MCF-7 were 17.59% for AuNPs-GAL, 19.9% for GAL, and 3.52% for AuNPs (Figure 6).

**Figure 7:**
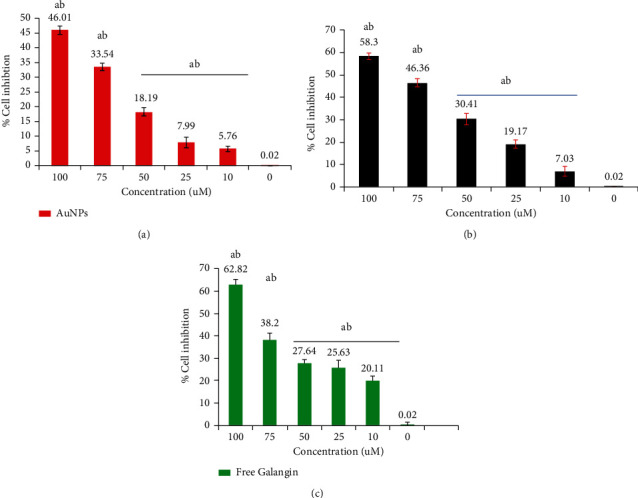
Cytotoxicity and growth cell inhibition determined by MTT assay on MDA-MB-231 human breast cancer cell growth exposed to (a) AuNPs, (b) AuNPs-GAL, and (c) GAL at different concentrations (10, 25, 50, 75, and 100 *μ*M). Statistical analysis indicates (a) significant difference compared to the control group and (b) significant difference between groups. Data are mean ± SD from three independent experiments.

MDA-MB-231 cell line growth inhibition results reveled that at 100 *μ*M, the highest cell inhibition was 58.3% for AuNPs-GAL, 62.82% for GAL, and 46.01% for AuNPs. At 10 *μ*M, the results were 5.76% for AuNPs, 7.03% for AuNPs-GAL, and 20.11% for GAL, respectively, as shown in Figure 7.

The conjugated AuNPs-GAL had much higher cytotoxic effects than did GAL individual. With a comparison of GAL concentration loaded on AuNPs and GAL alone, it is important to note that the decrease in cell viability suggested the ability to penetrate and accumulate inside the cells, stressing them out and ultimately leading to apoptosis [29]. Toxic effects of AuNPs might be attributed to the increase of proapoptotic p53 active members of the Bcl-2 family, Bax; this results in the outer mitochondrial membrane becoming permeable and releasing soluble proteins into the cytoplasm, where they trigger the intrinsic apoptosis pathway [34]. The lack of HER2 amplification, ER and PR gene expression, and changes in multidrug resistance (MDR) gene expression may have contributed to MDA-MB-231's low sensitivity [35]. Claudin-3 and Claudin-4 downregulation and MDR proteins' poor expression of the Ki-67 proliferation have been reported to have a significant role in drug resistance and drug accommodation [36].

### 3.7. Antiangiogenesis Studies

#### 3.7.1. Quantitative of *VEGF* and *ERKI* Gene by RT-PCR

**Figure 8:**
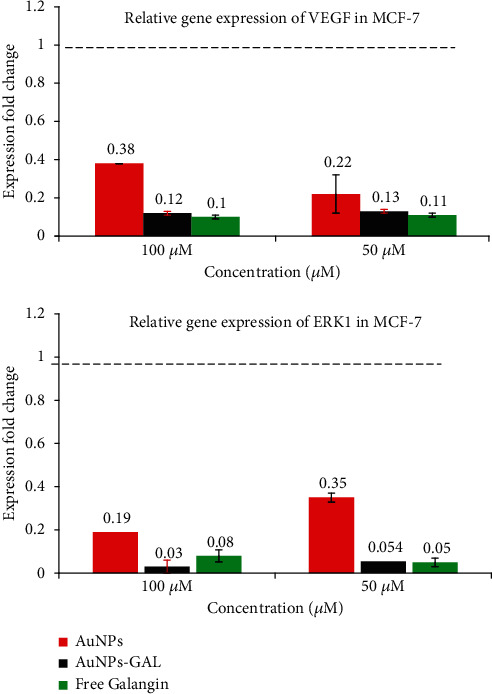
Relative gene expression from RT-PCR analysis in MCF-7 breast cancer cell line for VEGF and ERKI genes.

**Figure 9:**
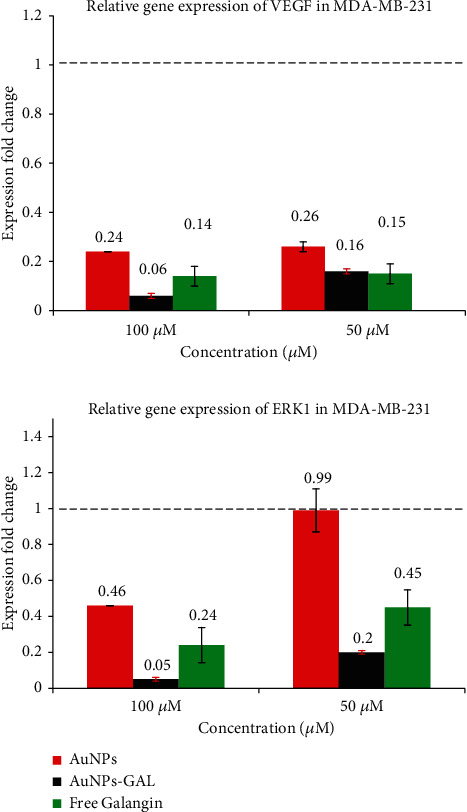
Relative gene expression from RT-PCR analysis in MCF-7 breast cancer cell line for VEGF and ERKI genes.

Exposure of both type of cells to AuNPs-GAL, AuNPs, and GAL significantly downregulated the expression of *ERK1* and *VEGF* genes in MCF-7 cells (Figure 8), while in MDA cells, the downregulation of interested genes for AuNPs-GAL, AuNPs, and GAL is shown in Figure 9. When compared to the MDA-231 cells, combined therapies for the MCF-7 cell produced more significant effects; this might be caused by HER2, PR, and ER in the MDA-231 cells [37]. When ERK1 gene expression is reduced, the cell cycle, tumor growth, angiogenesis, and migration are all inhibited. This is because the ERK1 signalling pathway is critical for controlling cyclin D1, migration, cell proliferation, and regulating cell growth [38]. One of the gold nanoparticle functions is to prevent phosphorylation of ERK1, VEGF, and VEGFR, which results in the inactivation of transcription factors for many genes; inhibition of the VEGF-A/VEGFR pathway can lead to inhibition of ERK1 phosphorylation, and this leads to the inhibition of the gene expression of many genes that contribute to vascular formation, migration, and cell cycle [39].

#### 3.7.2. Migration Assay

**Figure 10:**
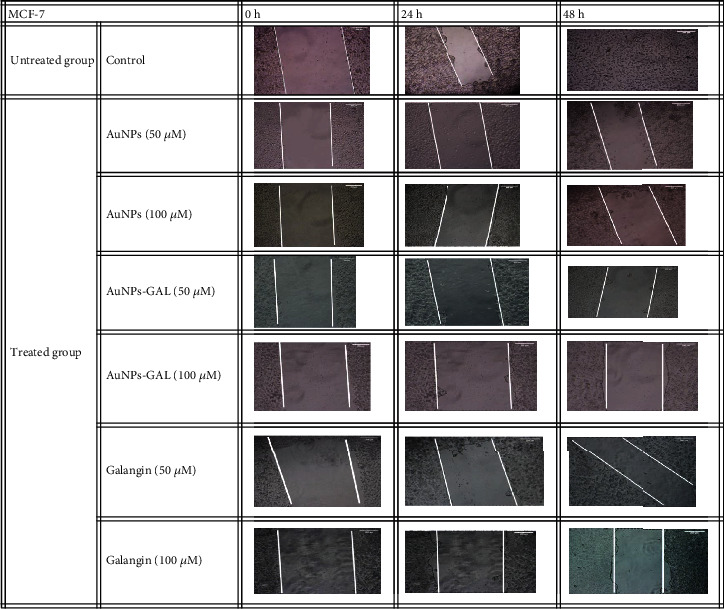
Scratch assay on MCF-7 cell line at different times (0, 24, and 48 h). Migration of cells in the treated groups was compared to the control group.

**Figure 11:**
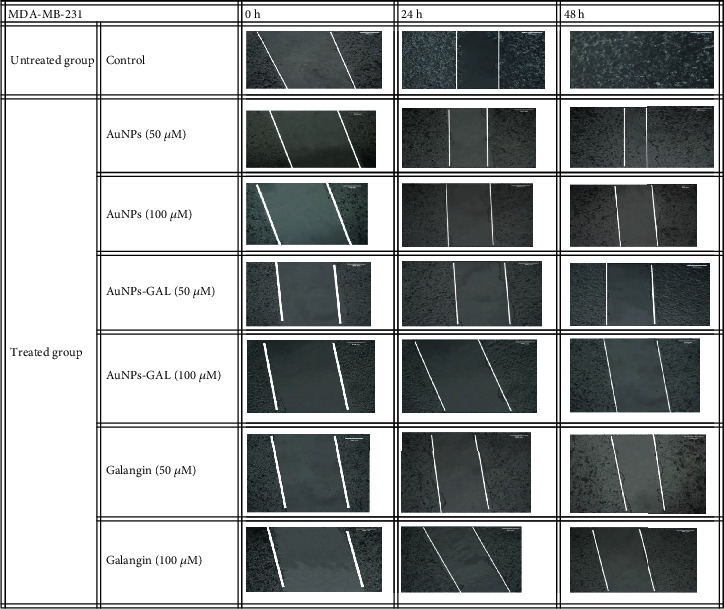
Scratch assay on MDA-MB-231 cell line at different timed (0, 24, and 48 h). Migration of cells in the treated groups was compared to the control group.

The effects of GAL, AuNPs, and AuNPs-GAL on migration of MCF-7 and MDA-231 cells were assessed by scratch/wound healing assay, and all results are summarized in Tables 2 and 3. Higher migration rate is seen in the control group; however, treatment with AuNPs, AuNPs-GAL, and GAL significantly decreased the migration of MCF-7 and MDA-231 cell lines (Figures 10 and 11), with a higher rate in the AuNP-GAL group.

It has been found that flavonoids can effect MMP-9 and MMP-2 which are key players in the processes of invasive metastasis; by destroying the structural component of the extracellular matrix, MMPs promote tumor invasion and metastasis [40]. Epithelial cells are stimulated by snail transcription factor in which mesenchymal genes are upregulated and cell-cell adhesion genes are downregulated [41].

#### 3.7.3. Chick Chorioallantoic Membrane (CAM) Assay

**Figure 12:**
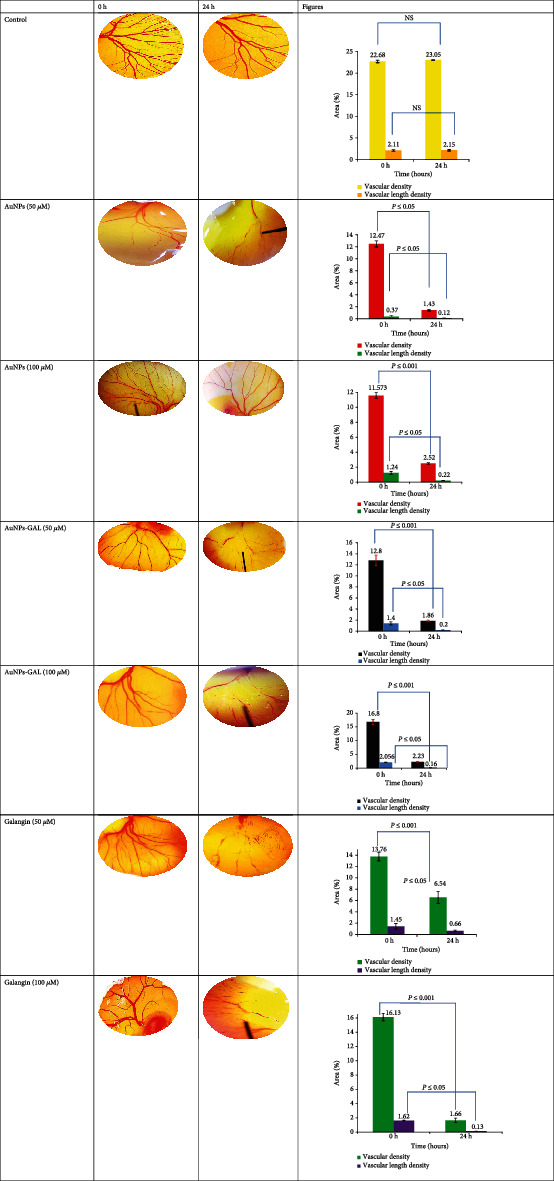
Images of implants maintained on the chicken embryo chorioallantoic membrane (CAM) for 24 h, soaked with control (not soaked); galangin, AuNPs, and AuNPs-GAL at 50 and 100 *μ*M. NS: not statically different.

Vascularized chorioallantoic membrane (CAM) assay in embryo chickens has been frequently utilized for angiogenesis studies. A typical vascular pattern with many branching was seen in the control group, as illustrated in Figure 12. Vascular density and vascular length density results were significantly (*P* ≤ 0.05) lower in the AuNP and AuNP-GAL groups than the control group; results of the CAM assay are summarized in the Table 4. Treatment with AuNPs-GAL was higher than that with AuNPs and GAL due to the synergistic effect of both AuNPs and GAL together on the inhibition of angiogenesis. According to research, AuNPs prevented the growth of new blood vessels and decreased the total vessel length and vessel functions. AuNPs were also found to decrease vascular permeability and density [42, 43]. Galangin acts as an anticancer and antiangiogenesis agent through the inhibition of CD44 and the suppression of EMT. Since CD44 plays a significant role in controlling the angiogenesis processes, which are factors in the initiation and progression of tumors, by preventing extracellular matrix, galangin reduced the growth, migration, and invasion of glioma cells [44].

## 4. Conclusions

This study is aimed at finding effective delivery strategies for getting sufficient levels of galangin to the tumor site through the use of gold nanoparticles. The findings of this study revealed that galangin and nanoparticles synergistically inhibit angiogenesis in two types of breast cancer cell lines (MCF-7 and MDA-MB-231) which was confirmed by scratch assay. Molecular results indicated significant downregulation of gene-meditated angiogenesis including *VEGF* and *ERKI* genes. In addition to inhibition of cell migration in monolayer cell line and formation of new blood vessels *in ovo*, the current nanostructure displayed a promising anticancer agent and antiangiogenesis agent through controlling *VEGF* and *ERKI* gene expression. Although to pinpoint the precise difference led to AuNP toxicity to cancer cells, additional research is necessary. Additional in vivo studies at various or even the same concentrations used are still required to determine the impact and understanding the underling mechanisms.

## Figures and Tables

**Table 1:** Primer sets for RT-PCR gene expression analysis (F: forward, R: reverse).

No.	Primer name	Seq.
1	GAPDH-F	5′-TCTCCTCTGACTTCAACAGCGAC-3′
2	GAPDH-R	5′-CCCTGTTGCTGTAGCCAAATTC-3′
3	VEGFA_exp-F	5′-TTGCTGCTCTACCTCCAC-3′
4	VEGFA_exp-R	5′-AAATGCTTTCTCCGCTCT-3′
5	ERK1_exp-F	5′-TGGCAAGCACTACCTGGATCAG-3′
6	ERK1_exp-R	5′-GCAGAGACTGTAGGTAGTTTCGG-3′

**Table 2:** Cell migration assay represented effects of AuNPs, AuNPs-GAL, and galangin on MCF-7 breast cancer cell. Different letters indicate significant difference at *P* ≤ 0.05 vs. control.

No.	Con. *μ*M/time	MCF-7 gap width distance (mean ± SD)			
		Control	AuNPs	AuNPs-GAL	Galangin
1	Control 0 h	806.38 ± 7.10^a^	806.38 ± 7.10^a^	806.38 ± 7.10^a^	806.38 ± 7.10^a^
2	100/24 h	419.28 ± 69.80^a^	683.97 ± 18.98^b^	772.37 ± 32.52^c^	643.34 ± 67.47^b^
3	100/48 h	133.63 ± 39.87^a^	625.39 ± 43.07^b^	672.71 ± 33.09^b^	526.37 ± 50.04^c^
4	Control 0 h	806.38 ± 7.10^a^	806.38 ± 7.10^a^	806.38 ± 7.10^a^	806.38 ± 7.10^a^
5	50/24 h	419.28 ± 69.80^a^	622.35 ± 53.88^b^	697.28 ± 47.81^b^	605.86 ± 33.57^b^
6	50/48 h	133.63 ± 39.87^a^	576.12 ± 86.46^b^	644.09 ± 32.83^c^	466.67 ± 55.87^c^

**Table 3:** Cell migration assay represented effects of AuNPs, AuNPs-GAL, and galangin on MDA-231 breast cancer cell. Different letters indicate significant difference at *P* ≤ 0.05 vs. control.

No.	Con. *μ*M/time	MDA-MB-231 gap width distance (mean ± SD)			
		Control untreated	AuNPs	AuNPs-GAL	Galangin
1	Control 0 h	723.42 ± 37.27^a^	723.42 ± 37.27^a^	723.42 ± 37.27^a^	723.42 ± 37.27^a^
2	100/24 h	372.67 ± 79.11^a^	486.42 ± 36.94^b^	689.12 ± 45.70^d^	598.47 ± 37.42^c^
3	100/48 h	96.99 ± 32.90^a^	457.58 ± 32.83^b^	575.60 ± 21.91^c^	535.87 ± 34.27^c^
4	Control 0 h	723.42 ± 37.27^a^	723.42 ± 37.27^a^	723.42 ± 37.27^a^	723.42 ± 37.27^a^
5	50/24 h	372.67 ± 79.11^a^	407.95 ± 35.20^b^	518.84 ± 66.26^c^	553.08 ± 39.70^c^
6	50/48 h	96.99 ± 32.90^a^	205.46 ± 34.39^b^	484.24 ± 46.43^c^	437.97 ± 58.71^c^

**Table 4:** CAM assay summary of vascular density. ^∗^Significant difference at *P* ≤ 0.05.

			Vascular density (%) mean ± SD			
No		Con. *μ*M/time	Control PBS	AuNPs	AuNPs-GAL	Galangin (GAL)
1	0 *μ*M	0 h	22.68 ± 0.29	—	—	—
		24 h	23.05 ± 0.08^∗^	—	—	—

2	50 *μ*M	0 h	—	12.47 ± 0.51	12.8 ± 0.95	13.76 ± 0.76
		24 h	—	1.43 ± 0.14^∗^	1.86 ± 0.13^∗^	6.54 ± 1.08^∗^

3	100 *μ*M	0 h	—	11.57 ± 0.39	16.8 ± 0.97	16.13 ± 0.52
		24 h	—	2.52 ± 0.1^∗^	2.23 ± 0.16^∗^	1.66 ± 0.3^∗^

## Data Availability

All data are presented within the article.
